# Kidney volume-to-birth weight ratio as an estimate of nephron endowment in extremely low birth weight preterm infants

**DOI:** 10.1038/s41598-024-64897-6

**Published:** 2024-06-18

**Authors:** Gabriele Villani, Pierluigi Zaza, Raffaella Lamparelli, Gianfranco Maffei

**Affiliations:** Neonatal Intensive Care Unit, Azienda Ospedaliero Universitaria Ospedali Riuniti, 71122 Foggia, Italy

**Keywords:** Medical research, Nephrology

## Abstract

In humans, nephrogenesis is completed by 32–36 weeks gestation, with a highly variable total number of nephrons, ranging from 200,000 to over 2 million. Premature birth disrupts the development and maturation of the kidneys, leading to a reduction in the final number of nephrons. Due to significant genetic variability in the number of nephrons among individuals, it is crucial to identify premature infants with fewer nephrons at birth as early as possible. These infants are more susceptible to developing renal failure with advancing age compared to those with a higher nephron endowment. Bedside ultrasound, an effective and non-invasive tool, is practical for identifying newborns with a lower nephron count. However, renal volume alone cannot reliably indicate the number of nephrons due to substantial variability at birth, influenced by gestational age when nephron maturation is incomplete. This variability in kidney volumes persists as newborns grow. In this observational study we hypothesize that the relationship between renal volume and birth weight may serve as an indicator of nephron endowment in premature infants with birth weight less than 1000 g. This finding could represent the basis for defining appropriate surveillance protocols and developing targeted therapeutic approaches.

## Introduction

Prematurity is the leading cause of infant death worldwide, and in most countries, the rate of preterm births is increasing with some variation between countries.

Thanks to advances in perinatal care, the survival of preterm infants has improved in recent decades. However, many of the surviving infants develop short- and long-term morbidity inversely proportional to gestational age (GA) and birth weight (BW). Therefore, there is a greater need to evaluate the outcomes in the short, medium, and long term^1^.

Mammalian kidney development begins at 5 weeks gestation, but nephrogenesis is a process not limited to intrauterine life. Nephrogenesis is completed by 32–36 weeks gestation, leading to a total number of nephrons ranging from 200,000 to over 2 million. This process continues for a period of 4–6 weeks after delivery in preterm infants^2,3^. Premature birth disrupts the development and maturation of all organs, including the kidneys, resulting in a reduction in the final number of nephrons and the development of abnormal and malfunctioning nephrons^4,5^.

In the fetus with intrauterine growth restriction (IUGR), the development of the renal system is further compromised due to the redistribution of blood flow to vital organs such as the brain^6,7^. The maturation time of nephrons is shorter in preterm infants with acute kidney injury (AKI)^8,9^. These initial conditions are aggravated by postnatal damage, further intensifying the nephron deficit^10^.

Corticosteroids, aminoglycosides, and xanthines have been identified as potential risk factors for renal damage. In addition to exposure to nephrotoxic drugs, preterm infants in the neonatal intensive care unit (NICU) may suffer from hypotensive crisis, severe infections, hypoxia, and hemodynamic impairment caused by patent ductus arteriosus (PDA) or its treatment, potentially leading to transient or permanent kidney failure^11–13^. These postnatal conditions impact the development of oligonephropathy in premature infants^14^.

Since Brenner et al. introduced the correlation between the low number of nephrons at birth and the resulting loss of nephrons through hyperfiltration and glomerulosclerosis, numerous studies have described the association between low birth weight and kidney size, kidney function, and blood pressure^15^. Compensatory glomerular hypertrophy and hyperfiltration have been associated with the development of hypertension, cardiovascular disease, and an increased likelihood of developing kidney failure, especially in adolescents and adults^16–18^.

As early as the 1960s, Damadian, Shwayri, and Bricker conducted histological and cytological analyses to detect the number of nephrons in the diseased kidneys of dogs^19^. In the following years, it was hoped that advances in imaging techniques could provide useful indications for recognizing premature infants with fewer nephrons at birth^20^. Relative volume measurement has also been effectively used in fetal studies as a ratio of kidney volume to estimated fetal weight^21–23^. Body weight seems to be less susceptible to error compared to other measures, such as body length, in the particular conditions faced by premature infants in the NICU.

Due to significant genetic variability in the number of nephrons among individuals, it is crucial to identify premature infants with fewer nephrons at birth as early as possible^24^. These infants are more susceptible to developing renal failure with advancing age compared to those with a higher nephron endowment in accordance with the Brennar hypothesis.

In this observational study, we investigate whether a correlation between renal volume and body weight can give indications about the nephron endowment in premature newborns with birth weight less than 1000 g.

## Results

We identified 46 extremely low birth weight (ELBW) premature infants born between March 2018 and March 2021 at NICU of Azienda Ospedaliero Universitaria Ospedali Riuniti, Foggia, Italy. Newborns with congenital anomalies were excluded from the study. Ultrasound examinations were used to calculate renal volume. The right and left renal volumes were summed to obtain the combined renal volume (CRV). The ratio of renal volume to body weight was used as an estimation of relative renal volume^25^. Renal ultrasounds were performed on all newborns; but, of the 46 infants identified, thirteen infants died during the study due to severe prematurity, two were excluded due to congenital malformations, and two were transferred to another hospital. Only 29 had traceable results and images and were therefore enrolled in the study. The first ultrasound examination was performed a week after birth (± 1 day) to allow for clinical stabilization of the newborns. Serial renal ultrasound examinations were then conducted at intervals of 14 days (± 1 day) up to 38–40 weeks post-menstrual age (PMA). For 5 newborns, the timing could not be exactly adhered to due to their precarious clinical conditions. Ultrasound follow-up was performed at 6, 12, 18, and 24 months PMA. Due to COVID-19 restrictions, only 10 newborns were able to complete ultrasound follow-up. A total of 224 ultrasound scans were performed.

Of the 29 ELBW preterm infants enrolled, 4 were classified as small for gestational age (SGA), while 25 were appropriate for gestational age (AGA), as determined by the percentile charts of the Italian Neonatal Study (INeS), which differentiate the neonatal growth curves based on sex and gestational age^26^. Among the participants, 9 were males and 20 were females. The gestational age at birth ranged from a minimum of 23 to a maximum of 31 weeks, with a median of 27 (interquartile range: 4). Similarly, the birth weight ranged from a minimum of 499 to a maximum of 998 g, with a median of 887 (interquartile range: 230).

Prophylaxis of hyaline membrane disease with prenatal Betamethasone was administered in 22 cases. Five newborns experienced asphyxia at birth, and 24 infants received at least one dose of endotracheal surfactant. In the NICU, all infants received xanthines and aminoglycosides. Eight infants received corticosteroids, and eight infants experienced hemodynamic impairment due to patent ductus arteriosus and/or its treatment. Additionally, ten infants had serious infections. Fluid and inotrope support have maintained systemic blood pressure within the normal range. Non-invasive ventilation was provided to all newborns, while 20 infants also received invasive mechanical ventilation. Three infants did not require oxygen supplementation therapy. Considering the maximum Fraction of Inspired Oxygen (FiO_2_) for each infant, the median FiO_2_ value for the 26 infants was 0.40 (interquartile range: 0.20), ranging from a minimum of 0.25 to a maximum of 1.00. For the definition of AKI, we used the modified Kidney Disease Improving Global Outcomes (KDIGO) criteria for newborns^3^. Serum creatinine (SCr) values were monitored during hospitalization, ranging from a minimum of 0.1 mg/dL to a maximum of 1.84 mg/dL, with a median of 0.58 (interquartile range: 0.65). One newborn had a maximum SCr of 1.53 mg/dL once, while another had a maximum SCr of 1.84 mg/dL once, without an increase. The data regarding urine output during hospitalization ranged from a minimum of 2.21 mL/kg/h to a maximum of 5.99 mL/kg/h, with a median of 3.8 (interquartile range: 1.31).

**Figure 1:**
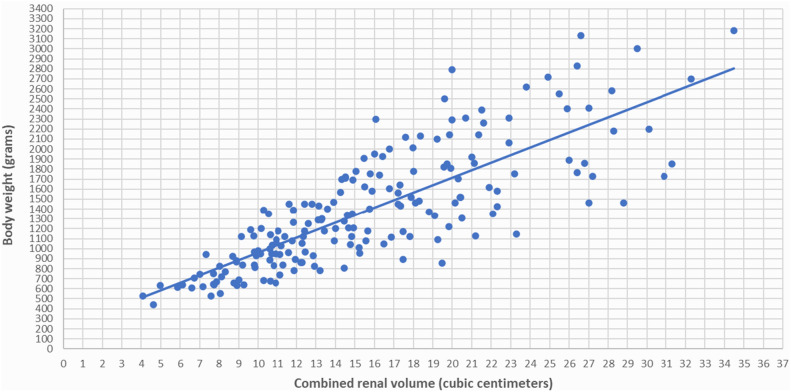
Relationship between combined renal volume (right renal volume + left renal volume) in cubic centimeters and body weight in grams of preterm infants with a birth weight of less than 1000 g from 1 week after birth to 38–40 weeks of post-menstrual age. Correlation coefficient r_s_ = 0.8321, p < 0.001.

**Figure 2:**
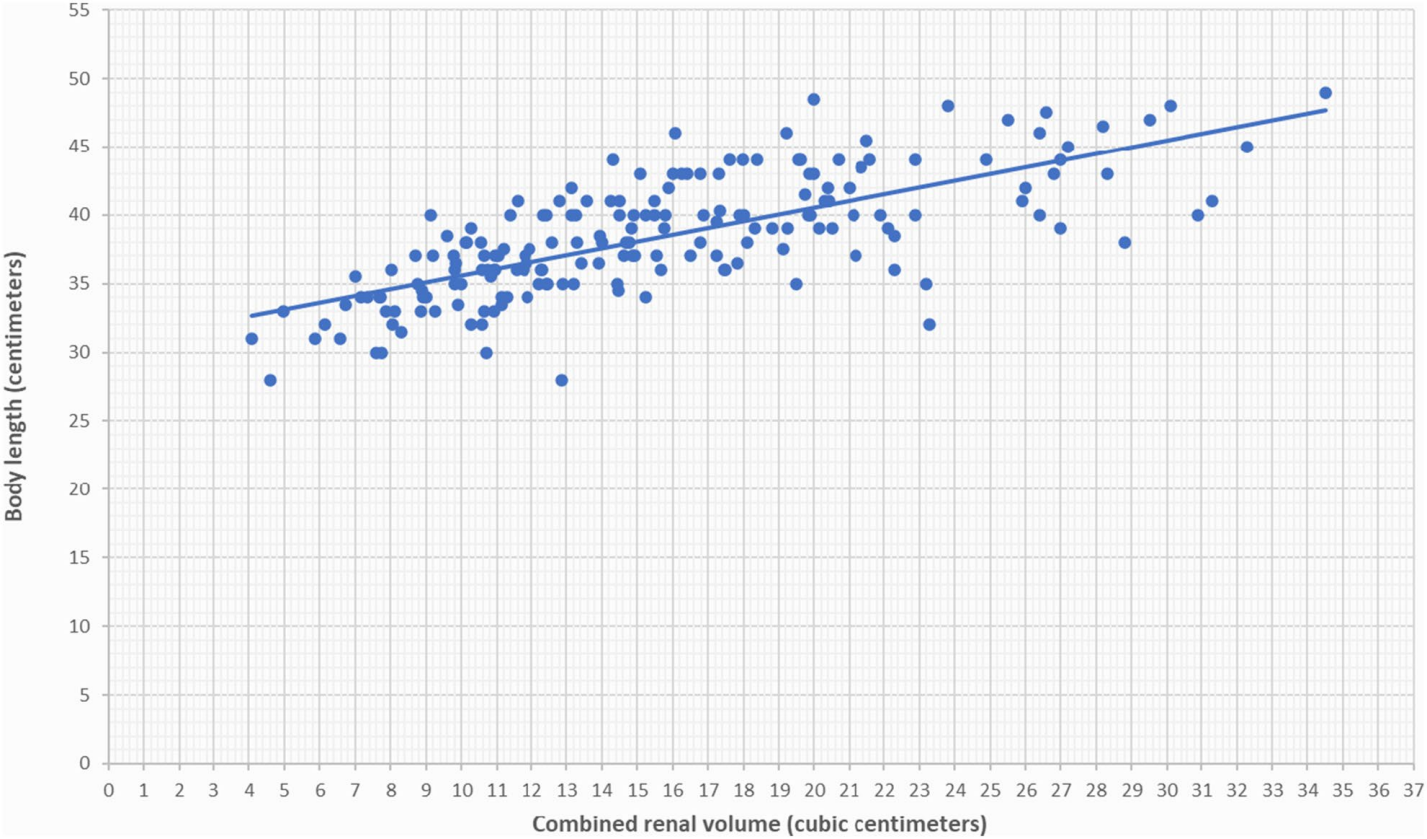
Relationship between combined renal volume (right renal volume + left renal volume) in cubic centimeters and body length in centimeters of preterm infants with a birth weight of less than 1000 g from 1 week after birth to 38–40 weeks post-menstrual age. Correlation coefficient r_s_ = 0.7339, p < 0.001.

**Figure 3:**
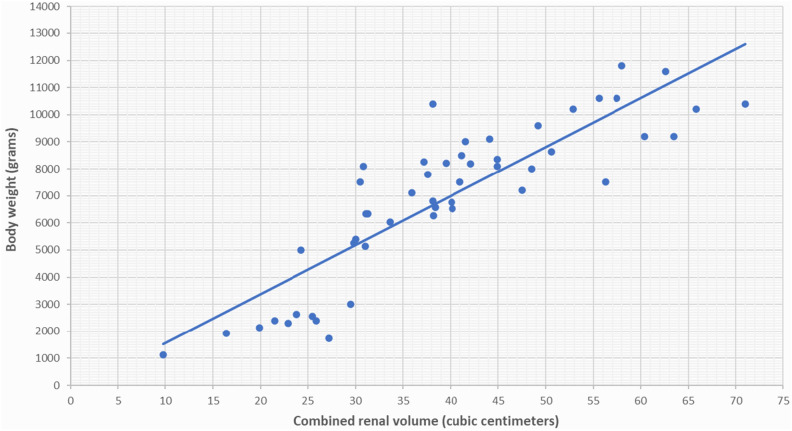
Relationship between combined renal volume (right renal volume + left renal volume) in cubic centimeters and body weight in grams of preterm infants with a birth weight of less than 1000 g from 38 to 40 weeks post-menstrual age to 24 months post-menstrual age. Correlation coefficient r_s_ = 0.8837, p < 0.001.

**Figure 4:**
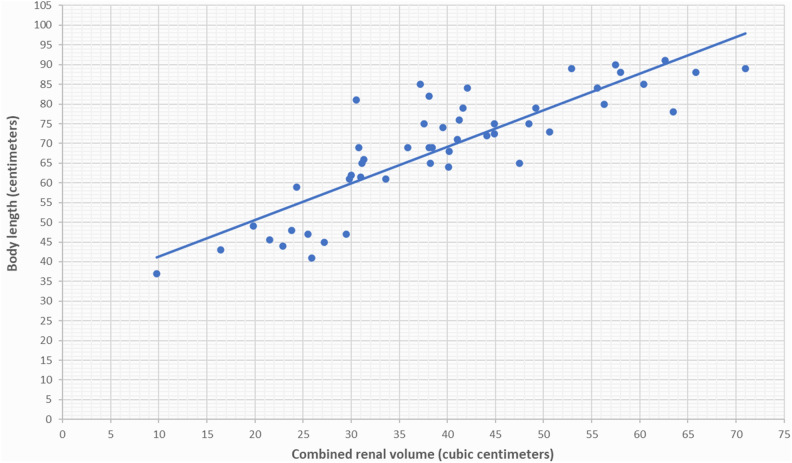
Relationship between combined renal volume (right renal volume + left renal volume) in cubic centimeters and body length in centimeters of preterm infants with a birth weight of less than 1000 g from 38 to 40 weeks post-menstrual age to 24 months post-menstrual age. Correlation coefficient r_s_ = 0.8493, p < 0.001.

Serial renal volumes, measured by ultrasonography from one week after birth to 38–40 weeks PMA and from 38–40 weeks PMA to 24 months PMA, were indexed to body weights in grams and to infant lengths in centimeters, which were recorded at the time of ultrasound scans. We observed a strong correlation between renal volume indexed by body weight and renal volume indexed by infant length for newborns both from one week after birth to 38–40 weeks of PMA (Figs. 1, 2) and from 38–40 weeks PMA to 24 months of PMA (Figs. 3, 4). However, the correlation coefficient was stronger for renal volume indexed by body weight. Specifically, from one week after birth to 38–40 weeks of PMA, the correlation coefficients were r_s_ = 0.8321, p < 0.001 for weight indexing compared to 0.7339, p < 0.001 for length indexing. From 38–40 weeks PMA to 24 months, the correlation coefficients were r_s_ = 0.8837, p < 0.001 for weight indexing compared to 0.8493, p < 0.001 for length indexing. We also considered the ratio of combined renal volume to body weight for each of the 29 newborns enrolled in our study at one week after birth, at 38–40 weeks PMA, and for 10 of them who continued follow-up at 6 months (mo) PMA, 12 mo PMA, 18 mo PMA, and 24 mo PMA. The ratio of combined renal volume to body weight at 38–40 weeks PMA tends to decrease (median 9.83; interquartile range 2.83) compared to that at one week after birth (median 11.73; interquartile range 2.91), with a p-value of 0.004701. In contrast, it remains almost constant between 6 and 24 months PMA (Table 1).

**Table 1:** Ratio of combined renal volume (CRV) (right renal volume + left renal volume, in cubic centimeters) to body weight (in grams) for each newborn at one week (wk) after birth, at 38–40 weeks post-menstrual age (PMA), at 6 months (mo) PMA, at 12 mo PMA, at 18 mo PMA, and at 24 mo PMA.

GA (wk)	CRV/body weight at one wk after birth	CRV/body weight at 38–40 wk PMA	CRV/body weight at 6 mo PMA	CRV/body weight at 12 mo PMA	CRV/body weight at 18 mo PMA	CRV/body weight at 24 mo PMA
24	10.41	10.75				
25	7.7	8.22				
25	10.8	7.84				
25	11.99	9.83	6.09	5.86	6.9	6.83
26	16.53	15.72	5.94	4.85	5.24	4.91
26	11.54	18.81				
26	14.16	17.86				
26	14.46	12.98				
26	11.73	16.92				
26	13.32	9.32				
27	11.27	8.5				
27	13.42	11.96				
27	7.89	8.47				
27	9.58	9.92	6.16	4.81	6.57	5.43
28	9.73	9.15				
28	13.82	10.79	6.6	5.38	5.19	5.4
29	10.59	10	5.55	5.84	6.07	7.51
29	12.06	9.27	4.95	5.55	5.13	6.45
30	12.11	8.65	4.86	4.92	5.47	4.87
30	11.76	11.92				
30	10.26	7.16				
30	11	8.52	5.68	5.6	4.83	5.14
30	11.49	9.97				
30	9.41	8.39				
30	11.97	9.08	6.04	5.06	4.07	4.51
30	14.21	10.85				
31	14.24	11.35				
31	12.07	8.3				
32	10.4	9	5.56	3.8	4.62	3.67
Median	11.73	9.83	5.81	5.22	5.22	5.27
IQR	2.91	2.83	0.54	0.75	1.24	1.58

*GA* gestational age in weeks (wk).

The ratio of combined renal volume to body weight at 38–40 weeks PMA is reduced (median 9.83; interquartile range—IQR 2.83) compared to that at 1 week after birth (median 11.73; interquartile range 2.91), with a p-value of 0.004701 (Mann–Whitney U test), while it remains almost constant between 6 and 24 months PMA.

## Discussion

In studies and clinical settings, kidney length and volume are commonly used as parameters to assess and monitor renal growth, especially in infants and children^27^. In a large study, Kim et al. evaluated the relationship between renal measurements and anthropometric indices and demonstrated that body weight has a closer correlation with renal volume than height, while kidney length is more closely related to the patient’s height^28^. We focused on renal volume measurements rather than kidney length because we deemed kidney length inadequate due to the approximately ellipsoidal shape of the kidney, which grows three-dimensionally. Although there is published literature on the use of body surface area (BSA) in newborns, the authors themselves admit that the clinical advantage lies in using only weight as a measure, since measuring body length for calculating BSA is often impractical due to the posture of newborns with reduced gestational age^29^. Future comparative studies may define a nomogram for surface area estimation for pediatric use, particularly given the increase in preterm births in modern society^30^.

Several studies have shown a correlation between gestational age, body weight at birth, and the number of nephrons^31–34^. Renal volume has been considered a likely indicator of nephron count. Singh et al. described in a specific Australian community, with participants aged 4.4 to 72.1 years, that smaller kidney volumes likely represent kidneys with a reduced number of nephrons^35^.

Genetic variants specific to certain populations, environmental factors during pregnancy such as maternal nutrition, exposure to toxic substances, and other environmental influences, as well as cultural habits and lifestyles that vary between different ethnic groups and geographical areas, may impact fetal kidney development; furthermore, socioeconomic differences may influence access to quality care and good nutrition, which in turn may affect fetal kidney development^36^. Therefore, renal volumes at birth of preterm infants may vary depending on geographic areas and ethnicities^37^. According to our study, even within the same gestational age range, renal volume varies considerably, as does body weight, among preterm infants one week after birth. Table 2 shows combined renal volume in cubic centimeters and body weight in grams, recorded at the time of ultrasound scans one week after birth, for the 29 ELBW premature infants, classified by gestational age in weeks. We believe, therefore, that renal volume alone is not a reliable indicator, and we hypothesize that an estimate of the nephron endowment at birth, which does not increase in number over time, can be obtained by indexing renal volume to body weight in this specific population of ‘children born too early’, since our data show a strong correlation between renal volume and body weight from one week after birth until at least 24 months of postmenstrual age. We also hypothesize that the tendency of the renal volume/body weight ratio to decrease from birth to 38–40 weeks, which remains almost constant between 6 and 24 months PMA, may be caused by the loss of nephrons due to pathogenic factors during hospitalization in the neonatal intensive care unit.

**Table 2:** Combined renal volume (right renal volume + left renal volume) in cubic centimeters and body weight in grams recorded at the time of ultrasound scans one week after birth for each preterm infant, categorized by their gestational age (GA) in weeks (wk).

GA (wk)	n		Min	Max	Median	Interquartile range
24	1	Combined renal volume			4.61	0
		Body weight			443	0
25	3	Combined renal volume	4.08	7.72	6.59	1.82
		Body weight	530	644	610	57
26	6	Combined renal volume	7.16	10.91	8.84	1.41
		Body weight	620	670	649.5	30
27	4	Combined renal volume	4.97	11.94	8.42	5.77
		Body weight	630	950	765	285
28	2	Combined renal volume	8.02	12.85	10.43	4.83
		Body weight	825	930	877.5	105
29	2	Combined renal volume	10.59	11.58	11.08	0.99
		Body weight	960	1000	980	40
30	8	Combined renal volume	7.75	12.22	9.51	1.96
		Body weight	640	950	863.5	68
31	2	Combined renal volume	9.84	12.30	11.07	2.46
		Body weight	815	864	839.5	49
32	1	Combined renal volume			10.73	0
		Body weight			1032	0

*n* number of preterm newborns with a birth weight of less than 1000 g.

In conclusion, chronic kidney disease is often silent, with symptoms typically emerging late, which complicates early diagnosis. Considering that prematurity is already a risk factor, premature infants with a lower genetic endowment of nephrons are more vulnerable and require increased monitoring. The aim of this study was to determine whether it is possible to estimate the nephron endowment at birth, which is genetically determined and highly variable from one individual to another. This aims to establish timely and collaborative nephrological follow-up, ensuring meticulous monitoring of premature infants throughout their lives.

Various techniques have been employed over the years to assess the number of nephrons in living individuals as accurately as possible. However, methods such as biopsy, Magnetic Resonance Imaging, and Computed Tomography are not suitable for premature infants at birth. Therefore, it was necessary to find an effective, non-invasive, and practical method. In the absence of standardized and agreed-upon reference values, and since the number of nephrons cannot be directly counted in vivo, the ratio of renal volume to body weight appears to provide a more reliable estimate of nephron endowment at birth. Although ultrasound is operator-dependent, it proves to be suitable for measuring renal volume at the bedside. Electronic scales integrated into modern incubators ensure more precise measurements than manual length measurements of newborns. Height measurement seems to be more suitable for taller age groups^38^.

The limitation of this observational study is its small sample size. Newborns with a birth weight of less than 1000 g constitute a very limited category, leading to small case numbers in single-center studies. We hope that this study will pave the way for a larger multicenter study aimed at establishing reference values for this category of newborns, defining adequate surveillance protocols, and developing targeted therapeutic approaches.

## Methods

The body weight measurement was taken using an electronic scale integrated into the incubator, as it minimizes measurement bias compared to measuring body length in such small infants. Ensuring proper hydration is essential for newborns with extremely low birth weight and is a fundamental aspect of good clinical practice. This is achieved through enteral and/or parenteral nutrition when necessary, coupled with continuous monitoring of the infants’ hydration levels. This makes body weight a parameter that can be utilized. The optimal methods or protocols for monitoring the onset of AKI remain unknown to date^39^. In our study, serum creatinine, quantified in milligrams per deciliter, was measured for all newborns on the third day after birth to avoid interference with maternal values. The assessment of creatininemia was repeated whenever clinical suspicion of AKI arose. Urine output was quantified by weighing the diaper or using a neonatal urine collection bag. The measurement, recorded daily, was expressed in milliliters per kilogram per hour. For non-invasive systemic blood pressure evaluation methods and reference values, the criteria indicated in Cloherty and Stark’s Manual of Neonatal Care—8th ed. were used^40^.

The ultrasound examinations were conducted using an ultrasound machine equipped with an abdominal convex ultrasound probe with a frequency of 5–8 MHz and preheated gel. An expert examiner performed all ultrasound examinations to minimize inter-observational errors. With the patient in the prone position, the sagittal plane was used to measure the length of the kidney by evaluating the maximum longitudinal distance cranio-caudally. The antero-posterior and transverse kidney diameters were measured perpendicularly in an axial plane where the kidney appeared symmetrically round. The images were sufficiently enlarged to ensure accurate measurements. The renal volume was calculated in cubic centimeters using the equation of an ellipsoid: volume = length × width × depth × 0.523^41^.

Parenteral and enteral nutrition were provided to the neonates according to the recommendations of the Neonatal Nutrition Task Force of the Italian Society of Neonatology, and the European Society for Pediatric Gastroenterology Hepatology and Nutrition (ESPGHAN).

During follow-up, the newborns underwent medical examinations in addition to renal ultrasounds. The clinical conditions of the children were good throughout the follow-up period.

The protocol was approved by the Ethical Committee at the Azienda Ospedaliero Universitaria Ospedali Riuniti, Foggia, Italy (Ref: 106, Opinion n. 54/CE/2018 approved during the session of 06/03/2018). The research was conducted in accordance with the Declaration of Helsinki and its subsequent amendments.

An informed consent document containing information about the study and consent for participation and publication has been obtained from the parents of all participants.

### Statistical analysis

All the analyses were performed using a commercially available package (SPSS, Rel 11.0 2002, SPSS Inc, Chicago, IL, USA). The Spearman’s rank correlation coefficient (r_s_) was used to measure the strength of the linear association between combined renal volume and body weight, as well as between combined renal volume and body length. An r_s_ value of 1 indicates a perfect positive correlation.

In order to ensure a reliable calculation of Spearman’s rank correlation coefficient, at least 5 ultrasound examinations were performed for each newborn, from one week after birth to 38–40 weeks PMA and from 38–40 weeks to 24 months PMA. P-value was considered significant for p < 0.05. Other data are presented as minimum value (min), maximum value (max), median, and interquartile range (IQR). The Mann–Whitney U test was used to compare the ratio of combined renal volume to body weight 1 week after birth, and the ratio of combined renal volume to body weight at 38–40 weeks PMA among the 29 enrolled infants.

## Data Availability

The datasets used and analysed during the current study are available from the corresponding author on reasonable request.
